# Using Patient-Reported Outcome Measures to Identify Mobility Limitation in Incurable Cancer

**DOI:** 10.1016/j.arrct.2026.100633

**Published:** 2026-05-27

**Authors:** Carmine Petrasso, Joanne Bayly, Stephen Ashford, Subashini Rajagopalan, Julie Whitney, Matthew Maddocks, INSPIRE consortium

**Affiliations:** a Faculty of Nursing, Midwifery and Palliative Care, Cicely Saunders Institute of Palliative Care, Policy and Rehabilitation, King’s College London, London, UK.; b Regional Hyper-Acute Rehabilitation Unit, London Northwest University Healthcare NHS Trust, Northwick Park Hospital, Harrow, UK.; c Department of Physiotherapy, The School of Life Course & Population Sciences, King’s College London, London, UK.

**Keywords:** Mobility, Neoplasms, Palliative care, Patient-reported outcomes, Rehabilitation

## Abstract

**Objectives:**

To (1) examine the relationship between a single-item and multicomponent patient-reported outcome measure (PROM) for mobility; (2) identify mobility profiles (PROM-defined patterns of mobility difficulty) using these measures; and (3) explore associations between demographic or clinical characteristics and mobility profiles.

**Design:**

Analysis of baseline data from an ongoing multinational trial of a rehabilitation intervention for people with incurable cancer.

**Setting:**

Oncology and palliative care outpatient services participating in an international rehabilitation trial.

**Participants:**

A consecutive sample of 211 adults with incurable solid tumors, Eastern Cooperative Oncology Group Performance Status 2-3, and a clinician estimated prognosis of ≥3 months. Of these, 206 had complete mobility data and formed the analytic sample.

**Main Outcome Measure(s):**

Mobility was assessed using the Integrated Palliative Care Outcome Scale (IPOS) mobility item and the World Health Organization Disability Assessment Schedule (WHODAS 2.0) mobility subdomain; higher scores reflect greater mobility limitation or difficulty. Secondary outcomes included WHODAS 2.0 self-care and participation scores and quality of life measured using the Functional Assessment of Cancer Therapy-General.

**Results:**

IPOS and WHODAS 2.0 mobility scores were positively correlated (ρ=0.650; 95% CI, 0.55-0.74; *P*<.001). Five mobility profiles were identified (Silhouette≈0.6), each defined by a single IPOS score (0-4) and WHODAS 2.0 mean scores ranging from 8.3 (SD, 3.7) to 19.3 (SD, 3.9). These groups primarily reflected a graded spectrum of mobility difficulty rather than distinct patterns. WHODAS 2.0 self-care, participation, and Functional Assessment of Cancer Therapy-General scores did not significantly differ by mobility profile. However, younger patients and those using mobility devices were represented more in mobility profiles with greater perceived mobility difficulty.

**Conclusions:**

A single-item PROM identified broad functional limitations and may help identify mobility profiles among people with incurable cancer. These findings support the utility of the IPOS mobility item as a tool that may help clinicians screen for difficulties with mobility and determine when detailed assessment or rehabilitation input is warranted. The PROM-defined mobility profiles should be interpreted as exploratory stratification and not as independently validated clinical subtypes. Further studies should validate the mobility profiles’ stability and clinical usefulness in longitudinal cohorts.

Mobility loss is a common feature of incurable cancer, affecting an estimated 50%-65% of people, though prevalence varies by diagnosis, disease stage, and care setting.^1^^,^^2^ Mobility loss contributes to reduced independence, increased hospitalization, treatment intolerance, and diminished quality of life, and places substantial demands on caregivers and healthcare services.^3^ Mobility difficulties arise from disease burden, treatment toxicity, and cumulative symptom effects,^4^ yet these needs often remain under-recognized in routine care.^5^ Mobility has been defined by Webber et al^6^ as “the ability to move oneself (either independently or with assistance) within environments that expand from the home to the community and beyond.”^6^ It is shaped by clinical, psychosocial, and environmental factors, fluctuates over time, and is a key marker of functional status.^6^ Early identification of mobility loss is therefore essential, as evidence indicates that timely rehabilitation may support functional ability, limit deconditioning, and reduce the consequences of progressive symptoms.^7^, ^8^, ^9^, ^10^

Use of validated performance-based assessments such as the 6-minute walk test is valuable when the goal is to assess physical capacity or functional reserve, or treatments to improve mobility performance.^11^ However, in oncology and palliative care settings, logistical constraints and an individual’s symptom burden may limit their feasibility or acceptability.^12^ In such contexts, patient-reported outcome measures (PROMs) offer a less burdensome alternative that reflects daily-life challenges more directly, and can identify functional difficulties that performance tests may miss.^13^ Two PROMs relevant to oncology and palliative care are the Integrated Palliative Care Outcome Scale (IPOS),^14^ and the 36-item World Health Organization Disability Assessment Schedule (WHODAS 2.0).^15^ IPOS is widely used in palliative care to assess symptom burden, including a single item on mobility.^14^ Despite its brevity, the IPOS mobility item reflects a key patient priority. In one study of 1540 people with advanced illness, poor mobility was rated as the top concern by 64.4% of participants.^16^ Another study found it to be the second most distressing symptom among 376 adults receiving palliative care.^17^ In contrast, the WHODAS 2.0 is a more comprehensive 36-item functional assessment tool, validated across a wide range of clinical populations, that includes a 5-item mobility subdomain.^15^^,^^18^

Practical considerations are equally important with PROMs, as mobility assessment must be feasible within clinical workflows and minimize the burden placed on patients.^19^ In this context, a single-item measure such as the IPOS mobility question may offer a low-burden way of capturing mobility difficulty and identify when a more detailed assessment is warranted.^17^ Demonstrating that this item captures similar functional constructs to the WHODAS 2.0 mobility subdomain may support its use for screening purposes. Therefore, this study aimed to: (1) examine the relationship between a single-item and multicomponent PROM of mobility; (2) identify mobility PROM-defined mobility profiles using these tools; and (3) explore associations between demographic or clinical characteristics with these mobility profiles.

## Methods

### Study design

A cross-sectional analysis of baseline data collected as part of an ongoing multinational trial evaluating a rehabilitation intervention for people with incurable cancer.^20^ The manuscript was written in accordance with the Strengthening the Reporting of Observational Studies in Epidemiology checklist.^21^

### Participants

Participants were adults (≥18y) with a diagnosis of incurable solid cancer, a clinician-estimated prognosis of >3 months, and an Eastern Cooperative Oncology Group (ECOG) performance status of 2 or 3. People with hematological malignancies or those currently receiving specialist oncological rehabilitation were excluded from the trial. The analysis included participants recruited between May 24, 2024 and May 27, 2025.

### Measures

Mobility was assessed using 2 PROMs: IPOS and WHODAS 2.0. IPOS evaluates how symptoms have affected the individual over the previous seven days on a single item with a scale from 0 (not at all) to 4 (overwhelmingly).^14^ IPOS is commonly used in people with serious and life limiting illness and was analyzed as an ordinal variable.^22^ The WHODAS 2.0 mobility subdomain comprises five items assessing: difficulty with standing for long periods, standing up from sitting, moving around inside the home, leaving the house, and walking long distances.^15^ Each item is rated on a 5-point Likert scale ranging from “no difficulty” to “extreme difficulty or cannot do,” resulting in a total score between 5 and 25. Higher scores reflect greater functional limitation, with scores of 6 or higher indicating a mobility limitation.^15^ WHODAS 2.0 mobility scores were treated as continuous variables, consistent with psychometric evidence showing that the summed scores satisfy assumptions for continuous data and offer greater statistical power than categorical approaches.^23^

In addition to mobility, 2 other WHODAS 2.0 subdomains were examined: self-care that assesses washing, dressing, feeding, and staying alone, and social participation that captures community involvement, emotional and financial impact. Both domains are scored in the same way as the WHODAS 2.0 mobility subdomain, with higher scores indicating greater difficulty.^15^ Quality of life was measured using the Functional Assessment of Cancer Therapy-General (FACT-G), a validated 27-item questionnaire assessing physical, emotional, social, and functional well-being in people with cancer.^24^ Total scores range from 0 to 108, with higher scores reflecting better overall quality of life.^24^

### Statistical analysis

All analyses were performed using IBM SPSS Statistics version 29.0 (IBM Corp., Armonk, NY)^a^ using complete case analysis with no imputation.^25^ Statistical significance was set at *P*<.05. Missing data were summarized for all covariates and secondary outcomes. Given the low proportion of missingness, analyses were conducted using complete-case methods. The primary analyses included participants with complete IPOS and WHODAS mobility data, whereas secondary analyses were based on available data for the variables included in each model.

Descriptive statistics were used to summarize IPOS and WHODAS 2.0 mobility scores, including means, SDs, and distributional checks using histograms. Spearman’s rank correlation was used to assess the relationship between IPOS and WHODAS 2.0 mobility scores, given the ordinal nature of the IPOS item and non-normality in WHODAS 2.0 distributions.

In this study, “mobility profiles” refers to empirically derived strata of participants who share similar levels of mobility limitation based on PROM data. This usage aligns with prior research applying cluster analysis to identify clinically meaningful functional or symptom-based subgroups in advanced illness.^26^, ^27^, ^28^ To explore whether the combination of IPOS and WHODAS 2.0 mobility scores could differentiate mobility profiles, a TwoStep cluster analysis was conducted using both measures as inputs. TwoStep clustering was chosen because it accommodates mixed variable types and automatically estimates the optimal number of clusters.^29^ Cluster quality was evaluated using the silhouette coefficient, with values ≥0.5 interpreted as indicating good separation.^30^ Cluster stability was evaluated through a sensitivity analysis using a randomly selected 80% subsample of participants. The TwoStep clustering procedure was repeated using the same input variables (IPOS mobility and WHODAS 2.0 mobility scores), and the resulting cluster number and score distributions were compared with those derived from the full sample.

Demographic and clinical characteristics were compared across mobility profiles using bivariate analyses. Variables considered included age (continuous), gender, mobility device use, living situation and residence type, all of which were self-reported via baseline case report forms. Primary cancer site and number of comorbidities, from a list of 24 predefined conditions, were recorded at baseline. Spearman’s rank correlation tested the relationship between age and comorbidity burden. A multinomial logistic regression was then conducted to identify independent predictors of mobility profiles, including all demographic and clinical variables. Cancer sites were grouped into broader categories to avoid small cell counts and improve model stability. Multicollinearity among predictors was assessed using variance inflation factors. To explore whether functional domains and quality of life differed across mobility profiles, 1-way analyses of variance (ANOVAs) were used to compare WHODAS 2.0 self-care, participation, and FACT-G scores. In addition, associations between IPOS mobility scores and these domains were examined using 1-way ANOVAs to assess whether higher perceived mobility impact was linked to greater difficulty in self-care, participation, or lower quality of life. These comparisons were interpreted cautiously given shared PROM methodology and potential construct overlap.

### Ethical considerations

The original trial received ethical approval from the London-Southeast Research Ethics Committee (REC reference: 23/LO/0991) in December 2023, and all participants provided written consent. In accordance with guidance from the UK Health Research Authority ethics decision tool, this analysis of anonymized, pre-existing trial data did not require additional ethical approval or consent procedures.^31^

## Results

### Sample characteristics

A total of 211 participants were enrolled in the study, of whom 206 (97.6%) provided complete data for both the IPOS and WHODAS 2.0 mobility measures and were included in this analysis (table 1). The mean (SD) age of the sample was 67.9 years (11.4), with a range of 28 to 91 years. Over half of the participants were female (n=112, 54.4%), and the large majority were living in a private residence (n=202, 95.7%). The most common cancer types were gastrointestinal (n=56, 27.2%), genitourinary (n=36, 17.5%), and thoracic (n=34, 16.5%). The number of comorbid conditions ranged from 0 to 5, with a median of 1. IPOS mobility scores spanned from 0 to 4 but were skewed toward higher levels of symptom burden: the median score was 2 (IQR, 1-3), and the mode was 2 (supplemental fig S1). WHODAS 2.0 mobility subdomain scores ranged from 5 to 25, with a mean of 13.8 (±5.1) and a median of 14 (supplemental fig S2). Missing data were minimal for demographic and clinical variables but higher for some secondary outcomes, particularly WHODAS participation (supplemental table S1).

**Table 1:** Participant characteristics

Characteristics	Participants (n=206)
Age, mean ± SD, min-max	67.9±11.4, 28-91
Sex, n (%)	
Female	112 (54.4)
Male	94 (45.6)
Cancer type, n (%)	
Gastrointestinal and hepatobiliary	56 (27.2)
Genitourinary	36 (17.5)
Thoracic	34 (16.5)
Breast	27 (13.1)
Gynecological	19 (9.2)
Head and neck	8 (3.9)
Other	22 (10.7)
Missing	4 (1.9)
Number of comorbidities, n (%)	
0	89 (43.2)
1	69 (33.5)
2	23 (11.2)
3+	25 (12.1)
Living, n (%)	
With spouse or other	154 (74.8)
Alone	49 (23.8)
Missing	3 (1.5)
Residence, n (%)	
Private	198 (96.1)
Institutional (care home/hospice)	1 (0.5)
Other (sheltered housing, hostel, refuge)	5 (2.4)
Missing	2 (1.0)
Education, n (%)	
No education	3 (1.5)
Primary	2 (1.0)
Lower secondary	34 (16.5)
Upper secondary	78 (37.9)
Bachelor’s degree	45 (21.8)
Master’s degree or PhD	24 (11.7)
Missing	20 (9.7)

A TwoStep cluster analysis using IPOS and WHODAS 2.0 mobility scores identified 5 mobility profiles representing a graded spectrum of mobility limitation ranging from ‘no reported difficulties’ to ‘overwhelming difficulty’ (table 2). Each mobility profile was defined by a single, uniform IPOS mobility score, resulting in an IQR of zero. Cluster quality was evaluated using the Silhouette coefficient, which indicated good separation between groups (≈0.6) (supplemental fig 3). Cluster sizes ranged from 13.6% to 28.2% of the sample, and within-group dispersion for WHODAS mobility was 3.0-4.3, suggesting homogeneous levels of mobility difficulty within each mobility profile (table 2). Mobility profile labels were assigned according to increasing IPOS mobility scores and corresponding WHODAS 2.0 mean values, reflecting progressively greater perceived mobility difficulty. A sensitivity analysis using a randomly selected 80% subsample (n=164) replicated the 5 mobility profiles, with comparable IPOS and WHODAS 2.0 score distributions and no change in cluster number (supplemental table S2). Cluster membership showed perfect agreement between the full sample and subsample analyses (Rand Index=1.00), indicating no reclassification of participants between mobility profiles.

**Table 2:** Mobility profiles derived from cluster analysis of IPOS and WHODAS 2.0 mobility scores

Mobility Profile	Label	N (%)	IPOS Score	WHODAS 2.0, Mean ± SD
1	No mobility difficulty	46 (22.3)	0	8.3±3.7
2	Mild mobility difficulty	35 (17.0)	1	11.4±4.3
3	Moderate mobility difficulty	58 (28.2)	2	13.1±3.0
4	Severe mobility difficulty	39 (18.9)	3	15.9±4.2
5	Overwhelming mobility difficulty	28 (13.6)	4	19.3±3.9

**Fig 1:**
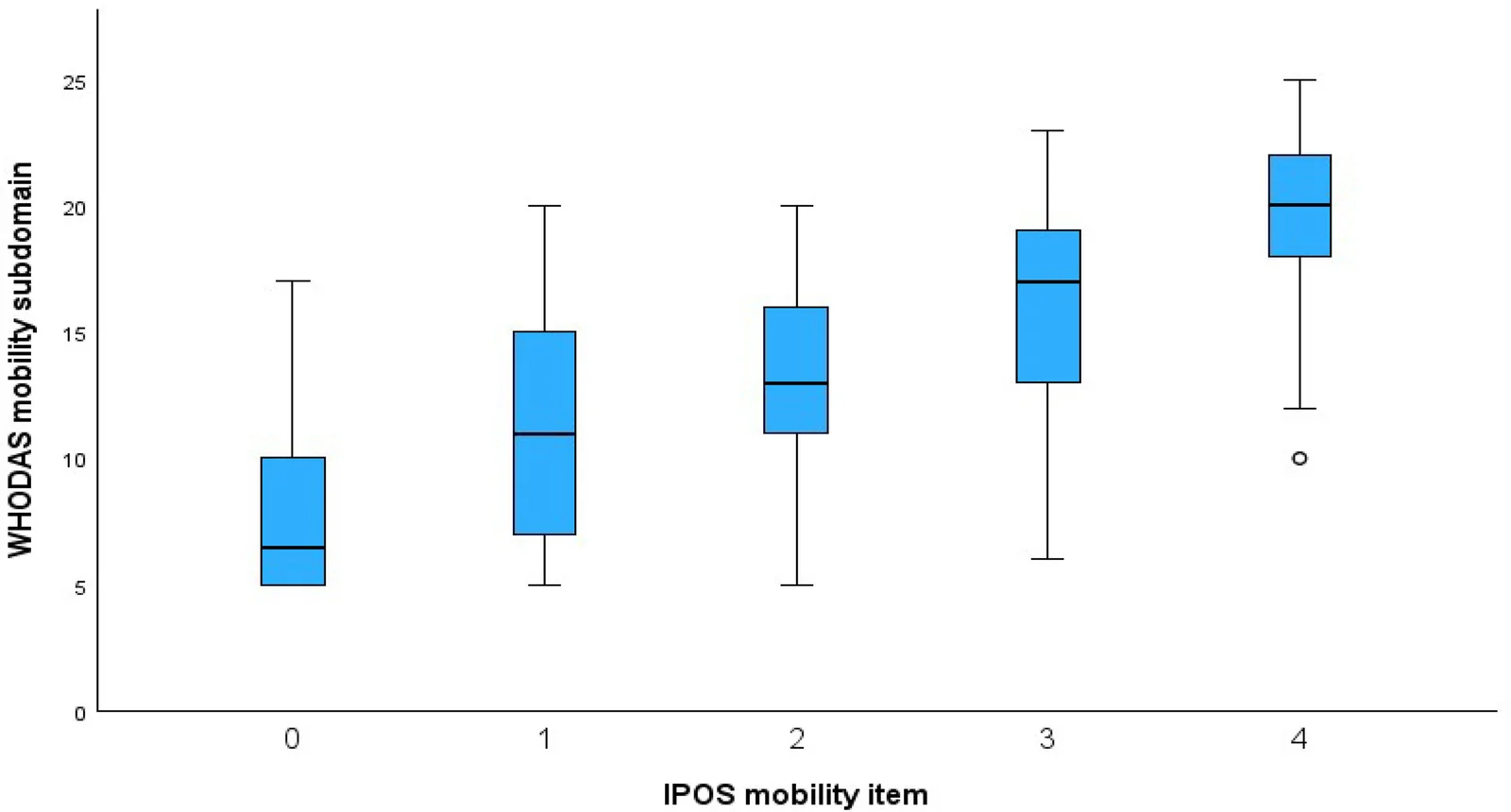
Boxplot showing the distribution of WHODAS 2.0 mobility subdomain scores stratified by IPOS mobility item responses.

There was a positive association between IPOS and WHODAS 2.0 mobility scores (ρ=0.65; 95% CI, 0.55-0.74; *P*<.001) (supplemental table S3). This relationship is illustrated in figure 1, which shows a stepwise increase in median WHODAS 2.0 mobility scores across all IPOS scores. Participants reporting no perceived mobility impact (IPOS=0) had a median WHODAS 2.0 mobility score of 6.5 (IQR 5.0-10.0), whereas those rating mobility as overwhelmingly affected (IPOS=4) had a median WHODAS 2.0 mobility score of 20.0 (IQR, 17.0-21.0).

**Fig 2:**
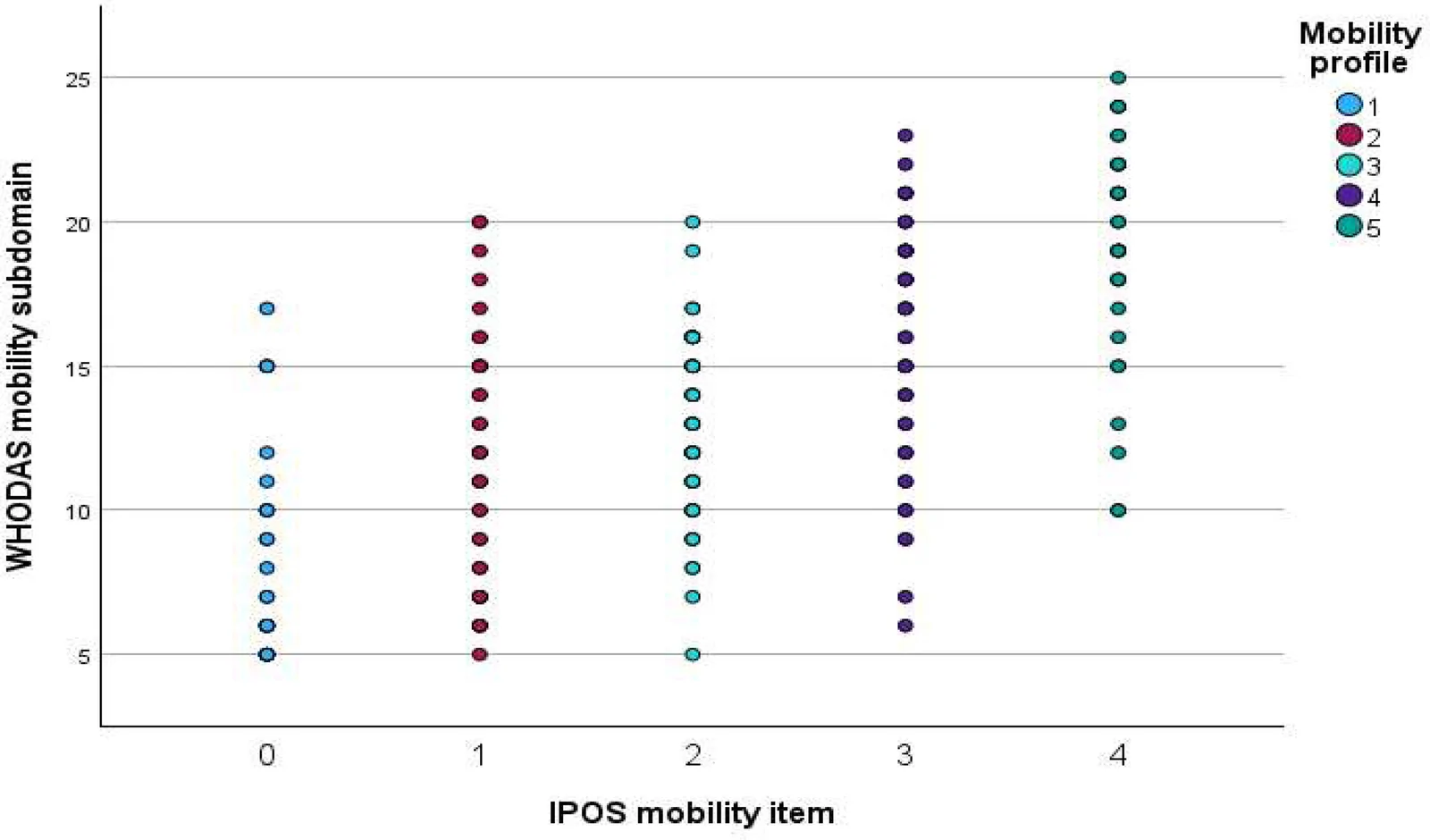
Scatterplot of WHODAS 2.0 mobility scores by IPOS response, colored by mobility profile.

Figure 2 illustrates the relationship between IPOS mobility scores and WHODAS 2.0 mobility subdomain scores, with each point color coded by mobility profile. As IPOS scores increase from 0 to 4, WHODAS 2.0 mobility scores also rise, indicating greater perceived mobility limitation.

Table 3 summarizes demographic and clinical characteristics across mobility profiles. Age and mobility device use differed significantly between groups, whereas no significant differences were observed for gender, living situation, residence type, or cancer site. Checks for multicollinearity indicated no overlap between predictors (all variance inflation factors<1.2).

**Table 3:** Participant characteristics by mobility profile

Characteristic	No Difficulty (n=46)	Mild Difficulty (n=35)	Moderate Difficulty (n=58)	Severe Difficulty (n=39)	Overwhelming Difficulty (n=28)	Test Statistic (*P* Value)
Age, mean ± SD	73.1±8.8	69.3±12.6	68.5±10.1	65.2±11.9	64.8±12.4	ANOVA: F_4,201_=2.998 (*P*=.02)
Sex, n (%)						χ²(4)=2.38 (*P*=.67)
Female	24 (52.2)	20 (57.1)	32 (55.2)	21 (53.8)	15 (53.6)	
Male	22 (47.8)	15 (42.9)	26 (44.8)	18 (46.2)	13 (46.4)	
Living situation, n (%)						χ²(4)=5.47 (*P*=.71)
With partner/other	36 (78.3)	29 (82.9)	45 (77.6)	31 (79.5)	24 (85.7)	
Alone	10 (21.7)	6 (17.1)	13 (22.4)	8 (20.5)	4 (14.3)	
Residence, n (%)						χ²(4)=2.83 (*P*=.95)
Private home	45 (97.8)	35 (100)	56 (96.6)	38 (97.4)	24 (96.0)	
Institutional/other	1 (2.2)	0	2 (3.4)	1 (2.6)	1 (4.0)	
Mobility device use, n (%)						χ²(4)=32.28 (*P*<.001)
None	36 (78.3)	23 (65.7)	29 (50.0)	11 (28.2)	8 (22.9)	
Yes	10 (21.7)	12 (34.3)	29 (50.0)	28 (71.8)	27 (77.1)	
Cancer site, n (%)						χ²(12)=13.89 (*P*=.31)*
GI/hepatobiliary	9 (19.6)	14 (40.0)	15 (25.9)	14 (35.9)	4 (14.3)	
Lung/thoracic	5 (10.9)	8 (22.9)	11 (19.0)	4 (10.3)	6 (21.4)	
Genitourinary	5 (10.9)	8 (22.9)	12 (20.7)	6 (15.4)	5 (17.9)	
Breast	3 (6.5)	1 (2.9)	7 (12.1)	11 (28.2)	5 (17.9)	
Other	24 (52.2)	4 (11.4)	13 (22.4)	4 (10.3)	8 (28.5)	

⁎ Cancer sites were collapsed into 5 categories due to small cell sizes.

Post hoc tests (n=206) revealed that participants in “no mobility difficulty” were significantly older than those in “severe mobility difficulty” (mean difference=7.9y; *P*=.03) and “overwhelming mobility difficulty” (mean difference=8.3y; *P*=.03). No other age differences between mobility profiles reached statistical significance. A Spearman’s rank correlation indicated a nonsignificant inverse relationship between age and comorbidity total (ρ=–.125), suggesting a trend toward higher comorbidity burden in younger participants. However, this association was not statistically significant (*P*=.07), and comorbidity burden appeared relatively consistent across age groups.

Use of mobility devices increased progressively across mobility profiles, from 22.9% in the “no mobility difficulty” group to 77.1% in the “overwhelming mobility difficulty” profile. Although overall cancer type distribution did not significantly differ between mobility profiles, an exploratory ordinal trend was detected (*P*=.008), suggesting a possible pattern in the distribution of cancer types across mobility profiles, with certain cancer sites appearing more frequently in groups with greater mobility difficulty (supplemental fig S4).

Descriptive analysis of the WHODAS 2.0 self-care (n=201; mean=8.0; SD=3.6) and participation (n=186; mean=21.2; SD=6.1) subdomains showed numerically lower scores than the mobility subdomain (mean=13.8; SD=5.1), suggesting fewer reported difficulties in these areas. Mean self-care, participation, and FACT-G scores across mobility profiles are shown in supplemental table S4. Neither self-care nor participation scores differed significantly across mobility profiles (self-care: *P*=.61; participation: *P*=.84), nor across IPOS mobility scores (self-care: *P*=.70; participation: *P*=.77). These comparisons were interpreted cautiously, given shared PROM methodology and potential overlap in functional constructs. Mean FACT-G (n=211) scores also varied little across the 5 mobility profiles (supplemental table S4), and a 1-way ANOVA revealed no statistically significant differences in quality of life across profiles (*P*=.77). A similar pattern was observed when comparing FACT-G scores across IPOS mobility levels, with slightly lower quality of life reported among participants with greater perceived mobility difficulty (mean=50.3) compared with those reporting less difficulty (mean=51.8), although this difference was not statistically significant (*P*=.34; Cohen’s *d*=–0.15).

## Discussion

This study examined the relationship between a single-item and a multicomponent PROM for mobility and used these measures to derive 5 PROM-defined mobility profiles among people with incurable cancer. By applying cluster analysis to PROM data, the study contributes new evidence on variation in patient-reported mobility difficulty within a population where mobility loss is common but often insufficiently characterized.^26^ However, as the clustering inputs were 2 correlated mobility measures, the resulting groups primarily reflect a graded spectrum of severity rather than distinct mobility patterns, and should not be interpreted as clinical subgroups.

These findings build on previous work demonstrating that PROMs can identify clinically meaningful subgroups in advanced illness. For example, Kaufmann et al^26^ identified symptom-based PROM subgroups among oncology patients with palliative care needs, revealing heterogeneity in symptom burden. However, their approach focused on multisymptom profiles rather than mobility specifically and included patients with a wide range of performance statuses. This limits the use of their subgroups to inform mobility assessment or rehabilitation pathways. The present study extends this literature by isolating mobility as a distinct functional construct and identifying mobility profiles in a clinically homogeneous cohort. This allows a more targeted understanding of mobility variation and its relationship to demographic and clinical characteristics. Despite the cohort being restricted to ECOG performance status 2-3, WHODAS, and IPOS mobility scores showed wide variability. This indicates that ECOG, although widely used, may be too coarse a measure to capture meaningful levels of mobility difficulty.^32^^,^^33^ Patients classified within the same performance category may experience different levels of mobility difficulty, underscoring the added value of PROMs for identifying functional variation that clinician-rated performance status may not capture. Notably, the WHODAS 2.0 subdomains of self-care and participation had lower mean scores and narrower ranges than the mobility subdomain, suggesting fewer reported difficulties in these areas. However, the absence of statistically significant differences in self-care, participation, and FACT-G across mobility profiles warrants cautious interpretation. This pattern may reflect restricted variance within an ECOG 2–3 sample, behavioral or psychological adaptation, potential ceiling/floor effects in adjacent domains, or limited validity of PROM-defined mobility profiles. Notably, the self-care and participation domains showed narrower score distributions than the mobility subdomain in this cohort, suggesting reduced variability in these areas. Construct overlap across PROMs may also influence observed associations. In addition, mobility limitations may emerge earlier or be perceived as more disruptive than difficulties in other domains, which can sometimes be compensated for through environmental support or behavioral adaptation.^34^^,^^35^ Nonetheless, further work is needed to test whether mobility stratification differentiates functional domains in longitudinal samples.

A key finding was the inverse relationship between age and mobility profile, with younger individuals more frequently represented in profiles characterized by greater mobility limitation or difficulty. This pattern reflects observations in advanced lung cancer populations, where younger adults report higher disability despite comparable objective function.^36^ However, this association should be interpreted with caution, as it may reflect features of the study design and sample rather than a true underlying relationship. Factors such as the restricted ECOG range, sample composition, and the cross-sectional nature of the data may have influenced the observed pattern. Other potential explanations include younger adults typically engaging in wider life-space mobility and having responsibilities related to employment and childcare, meaning that functional losses disrupt a broader range of valued activities.^37^ They may also hold higher expectations for physical functioning and have lower tolerance for mobility loss, resulting in greater perceived difficulty.^37^ In contrast, older adults may normalize mobility limitations or recalibrate expectations, and may already have adopted adaptive strategies that mitigate the perceived impact of decline.^38^^,^^39^

Other clinical factors may also shape mobility trajectories. Certain cancer types, such as lung, gastrointestinal, and metastatic bone disease, are associated with symptom clusters such as breathlessness, cachexia, pain, or skeletal instability, all of which can impact mobility.^40^ As expected, mobility device use increased progressively across mobility profiles. However, despite 3-quarters of patients in palliative care settings receiving necessary mobility devices, unmet need remains a concern, with 1 in 4 still lacking access to required devices.^41^

The identification of mobility profiles in this study illustrates how PROMs can reveal variation in functional status among people with incurable cancer. Unlike clinician-rated tools, which may lack granularity, be subject to bias, and can only ever be a proxy measure, PROMs better reflect the person’s lived experience.^42^ For example, in a geriatric oncology study, >90% of patients rated as ECOG 0 or 1 had ≥1 functional difficulty identified through self-report or geriatric assessments.^43^ Although PROMs provide critical perspectives on perceived burden and functional mobility, performance-based assessments such as gait speed or timed sit-to-stand tests offer additional insights into capacity at a body systems level and may be useful in identifying subtle decline or monitoring response to intervention, although still may be less responsive than a PROM.^44^ Therefore, combining multiple assessment approaches may offer a more comprehensive understanding of mobility needs.^8^

Despite clear differences in mobility impairment across mobility profiles, overall quality of life scores did not differ significantly. This is perhaps unsurprising, as global quality of life reflects multiple dimensions of well-being, of which mobility is only 1 component. Patients may draw on psychological adaptation, resilience, or other resources to maintain perceived quality of life,^45^^,^^46^ meaning that changes in mobility may not be directly mirrored in overall scores. However, these findings should not be overinterpreted as evidence that mobility difficulty is unrelated to participation, self-care, or quality of life; rather, they may reflect measurement limitations, restricted variance, and the cross-sectional design.

### Clinical implications

This study highlights several potential uses of PROM-based mobility assessment in routine oncology and palliative care. The IPOS mobility item is already embedded in routine clinical practice across many palliative care services, particularly in the UK, where it forms part of the Outcome Assessment and Complexity Collaborative suite of standardized measures used to monitor patient needs and outcomes.^47^ As IPOS is already familiar to clinicians and routinely collected in many services, it offers a pragmatic initial screen for mobility concerns, helping identify patients who may require further mobility assessment or rehabilitation input without adding additional assessment burden.

The mobility profiles identified in this study offer a way of conceptualizing different levels of mobility difficulty that may inform clinical discussions about mobility needs and referral considerations. Although WHODAS self-care, participation, and FACT-G scores did not differ significantly across mobility profiles, this pattern suggests that mobility limitations may arise even when broader self-care, participation, or overall quality of life remain relatively preserved. This underscores the value of pairing mobility-specific PROMs with broader measures of function and well-being. PROM-based mobility assessment, therefore, represents a scalable and patient-centered approach to detecting emerging or fluctuating disability, supporting multidisciplinary team discussions, guiding referral decisions, and improving access to timely rehabilitation within palliative care settings.^48^

### Strengths and limitations

This study draws on a well characterized cohort of people with incurable cancer, enabling meaningful analysis of functional variation and influencing factors. TwoStep clustering enabled empirical stratification of mobility difficulty using PROM data, with the resulting mobility profiles reflecting graded levels of mobility difficulty rather than distinct clinical subgroups. However, the cross-sectional design limits interpretation of temporal change, and longitudinal research is needed to examine whether mobility profiles remain stable, evolve with disease progression, or predict clinical outcomes. In addition, the study relied solely on PROMs to characterize mobility, which prevented direct comparison with observed functional performance. As a result, it cannot be determined whether the IPOS mobility item reflects underlying physical capacity, perceived burden, or a combination of both. It is also important to recognize that the IPOS mobility item captures the extent to which mobility problems affect the individual, whereas the WHODAS 2.0 mobility subdomain assesses difficulty in performing specific activities. These measures therefore reflect related but distinct aspects of mobility, which may contribute to differences in how mobility limitation is reported and interpreted. The absence of an objective performance measure limits conclusions about how closely patient-reported mobility corresponds to physical performance. Selective use of brief, tolerable measures in future research, such as gait speed, may help clarify how perceived and measured mobility intersect. Moreover, the regression analyses should be interpreted cautiously and as exploratory, given the number of predictors relative to group sizes. Finally, PROM responses may be influenced by transient symptoms or fluctuating health, although such variability also affects performance-based measures.^49^

## Conclusion

This study demonstrates that the single-item IPOS mobility tool can reflect variation in functional limitation compared with the multicomponent WHODAS 2.0 mobility subdomain. A positive association between the 2 tools supports the utility of the IPOS mobility item as a screening measure. Using both tools, 5 PROM-defined mobility profiles were derived, representing a graded spectrum of perceived mobility difficulty. The overrepresentation of younger patients in mobility profiles characterized by greater difficulty with mobility challenges age-based assumptions and reinforces the importance of individualized assessment. Embedding PROM-based mobility phenotyping into routine care may help support early recognition of functional decline and support timely intervention. Future research should investigate whether these mobility profiles are stable over time, predict key outcomes, and how they relate to performance based functional measures.

## Supplier

a. IBM SPSS Statistics, version 29.0, IBM Corp., Armonk, NY.

## Disclosure

The investigators have no financial or nonfinancial disclosures to make in relation to this project.

## Appendix Supplementary materials

Image, application 1

Image, application 2

## Footnotes

Supplementary material associated with this article can be found, in the online version, at doi:10.1016/j.arrct.2026.100633.
