# Supplementary material for: Using Patient-Reported Outcome Measures to Identify Mobility Limitation in Incurable Cancer
Source: Arch Rehabil Res Clin Transl. 2026 May 27;8(3):100633. doi: 10.1016/j.arrct.2026.100633 (PMC13591189; doi:10.1016/j.arrct.2026.100633)
Supplement: Supplementary file 1 [file mmc1.docx]

**Supplementary Figure 1:** Distribution of IPOS scores at baseline

**Supplementary Figure 2:** Distribution of WHODAS 2.0 mobility scores at baseline

**Supplementary Table 1:** Missing data for covariates and secondary outcomes

| **Variable** | **Missing n (%)** | **N analysed** |
| --- | --- | --- |
| Age | 0 (0.0) | 211 |
| Gender | 0 (0.0) | 211 |
| Cancer site | 4 (1.9) | 207 |
| Mobility device use | 12 (5.7) | 199 |
| Residence (where living) | 2 (0.9) | 209 |
| Living situation | 3 (1.4) | 208 |
| Comorbidity total | 0 (0.0) | 211 |
| WHODAS participation | 25 (11.8) | 186 |
| WHODAS self-care | 10 (4.7) | 201 |
| FACT-G | 0 (0.0) | 211 |

**Supplementary Table 2:** Spearman’s correlation between IPOS mobility item and WHODAS mobility subdomain

|  | IPOS mobility item | WHODAS mobility subdomain |
| --- | --- | --- |
| IPOS mobility item | 1.00 | 0.65 (95% CI 0.55-0.74)* |
| WHODAS mobility subdomain | 0.65 (95% CI 0.55-0.74)* | 1.00 |

**p*<0.001, N=206

**Supplementary Figure 3:** Mobility profile summary showing model inputs, number of clusters, and silhouette-based quality assessment.

**Supplementary Table 3:** Sensitivity analysis of cluster stability: Comparison of WHODAS mobility scores in full sample and 80% subsample

| **Cluster** | **IPOS** | **WHODAS Mean – original (SD)** | **WHODAS Mean – subsample (SD)** |
| --- | --- | --- | --- |
| **1** | 0 | 8.25 (3.67) | 8.29 (3.59) |
| **2** | 1 | 11.44 (4.32) | 11.94 (4.18) |
| **3** | 2 | 13.05 (3.02) | 13.16 (3.31) |
| **4** | 3 | 15.87 (4.20) | 15.85 (4.15) |
| **5** | 4 | 19.31 (3.86) | 19.26 (3.90) |

**Supplementary Figure 4:** Distribution of primary cancer sites across mobility profiles
