# Supplementary material for: Using Patient-Reported Outcome Measures to Identify Mobility Limitation in Incurable Cancer
Source: Arch Rehabil Res Clin Transl. 2026 May 27;8(3):100633. doi: 10.1016/j.arrct.2026.100633 (PMC13591189; doi:10.1016/j.arrct.2026.100633)
Supplement: Supplementary file 2 [file mmc2.docx]

**Supplementary Table 4.** WHODAS self-care, WHODAS participation, and FACT-G scores across mobility profiles

| **Mobility profile** | **WHODAS self-care mean (SD)** | **WHODAS participation mean (SD)** | **FACT-G mean (SD)** |
| --- | --- | --- | --- |
| No mobility difficulty | 7.22 (3.05) | 20.09 (5.36) | 51.81 (8.61) |
| Mild mobility difficulty | 7.80 (3.65) | 20.49 (6.00) | 52.11 (8.71) |
| Moderate mobility difficulty | 8.23 (3.46) | 20.96 (6.41) | 50.87 (9.57) |
| Severe mobility difficulty | 8.42 (4.38) | 22.89 (6.22) | 50.88 (9.83) |
| Overwhelming mobility difficulty | 8.43 (3.47) | 22.00 (6.64) | 49.48 (11.51) |
